# Effectiveness of Different Diode Laser Wavelengths in Targeting *Enterococcus faecalis* Biofilm in Root Canal Treatment

**DOI:** 10.2174/0115748871367667250912111157

**Published:** 2025-10-07

**Authors:** Mohammed Al-Jaberi, Salman Sahab Atshan, Sonia Zouiten

**Affiliations:** 1 Department of Dentistry, College of Medicine Ibn Al Jazzar, University of Sousse, Sousse, Tunisia;; 2 Department of Dentistry, Al-Kunooze University College, Basrah, Iraq;; 3 Department of Medical Science, College of Dentistry, University of Basrah, Basrah, Iraq

**Keywords:** Diode laser, photoactivated disinfection, root canal disinfectant, *enterococcus faecalis* biofilms, sodium hypochlorite, endodontics

## Abstract

**Introduction:**

Disinfection of the root canal system is crucial for the effectiveness of root canal treatment. Lasers and photoactivated disinfection (PAD) have emerged as preferred methods for eliminating pathogens from the root canal.

**Methods:**

Sixty intact, freshly extracted adult human uniradicular mature teeth with a single root canal were collected. The crowns were removed, resulting in canals measuring 14 mm in length. The root canals were prepared, sterilized, and then inoculated with broth containing *Enterococcus faecalis (E. faecalis)*, followed by incubation for 30 days in an aerobic environment at 37°C. Biofilm formation was verified using a scanning electron microscope. The samples were randomly divided into six experimental groups (*n* = 10). Group 1 consisted of teeth treated only with distilled water. Group 2 teeth received 3% NaOCl and 17% EDTA as part of Conventional Chemomechanical Debridement (CCMD) but no additional treatment. Groups 3-6 also received CCMD followed by additional laser disinfection as follows: Group 3 underwent photoactivated disinfection (PAD) using riboflavin with a 450 nm laser; Group 4 underwent PAD using toluidine blue O (TBO) with a 635 nm laser; Group 5 underwent conventional laser endodontics (CLE) with an 808 nm laser; and Group 6 underwent CLE using triple wavelengths of 450 nm, 635 nm, and 808 nm.

**Results:**

The Kruskal-Wallis test revealed significant differences in colony-forming units (CFUs) among the groups after treatment (*p* < 0.001). Subsequent analysis showed that the difference in mean CFUs between the PAD groups and the CLE groups was not statistically significant. The group treated with the triple laser wavelength exhibited the lowest average CFUs/mL, while the distilled water group had the highest mean value.

**Discussion:**

The study confirms that diode laser-assisted disinfection significantly enhances bacterial reduction compared with conventional irrigation alone. Although PAD methods reduced *E. faecalis*, their effect was not statistically superior to conventional laser endodontics (CLE). The triple-wavelength diode laser group achieved the greatest bacterial reduction, likely due to the synergistic effects of thermal and photochemical interactions. These findings support the adjunctive use of laser disinfection to improve root canal decontamination, particularly when combined with chemomechanical preparation.

**Conclusion:**

This study demonstrates that combining an irrigating solution with a diode laser enhances the effectiveness of reducing pathogenic numbers.

## 1 INTRODUCTION

The primary reason for root canal failure is a bacterial infection that persists in specific anatomical regions, including isthmuses, lateral canals, recesses, and dentinal tubules, which are often difficult to access with instruments and antibacterial agents used in root canal therapy [1]. *Enterococcus faecalis (E. faecalis)* has been identified as the sole organism in root-filled teeth with periradicular lesions in certain instances [2].

The prevalence of *E. faecalis* in persistent periradicular lesions has been demonstrated to be significantly greater. Patients who have failed root canal therapy have a significantly higher likelihood of harboring *E. faecalis* compared to those with primary endodontic infections, with a nine-fold increase [3].

Apical periodontitis is an infection caused by biofilm. Biofilm provides a protective barrier for bacteria, shielding them from the host's immune system and enhancing their ability to withstand intracanal disinfection procedures. To develop new methods for disinfecting root canals, it is crucial to understand the pathogenicity of microorganisms present in the root canal biofilm [4]. The typical depth to which bacteria penetrate dentinal tubules is 500 μm [5]. One common bacterium that causes chronic apical periodontitis is *E. faecalis* [6]. After three weeks of incubation, its penetration can extend to greater depths, reaching 800-1000 μm in the dentinal tubules [7].

Diode laser light has a greater ability to penetrate dentin compared with typical chemical irrigants, reaching depths of up to 1100 μm. This phenomenon, known as the “light fog,” enables it to reach microorganisms trapped within the deeper layers of dentin [8].

Laser technology can be employed in two ways for endodontic treatment. The first method, known as conventional laser endodontics (CLE), involves directly exposing the dentin walls to laser irradiation. The second method, known as photoactivated disinfection (PAD) or laser-activated irrigation, utilizes lasers to irradiate or activate photoactive substances or irrigants. These approaches indirectly target the endodontic system, effectively achieving the desired clinical effect [9].

The effects of diode lasers on microorganisms can be classified into two categories: photo-thermal and photo-activated. The photo-thermal effect relies on an elevation in temperature, as heat causes damage [10].

Photoactivated disinfection (PAD) uses photosensitizers with antimicrobial properties, which are applied to the root canal and activated by specific wavelengths of light. The photoactive solution undergoes a photochemical reaction upon laser irradiation, leading to bactericidal effects through the release of singlet oxygen and reactive radicals. The dentin surface is not directly exposed to the laser, significantly reducing the risk of unwanted side effects [9].

The purpose of this research is to compare the effectiveness of different photosensitizers activated by diode laser at low laser wavelengths when used as photoactivated disinfection, and to compare it with other diode laser wavelengths as conventional laser endodontics (CLE) applications in conjunction with traditional disinfectants.

## 2 MATERIALS AND METHODS

### 2.1 Preparation of Samples

Ethical clearance for the study was granted by the Research Ethics Committee of the Faculty of Dentistry at Al-Kunooze University in Iraq (KDC 025). The study used extracted human teeth obtained from the Oral Surgery Clinic at the College of Dentistry, Al-Kunooze University. All teeth were extracted for orthodontic purposes, and informed consent was obtained from each patient for the use of their teeth in research. A total of sixty intact, freshly extracted adult human uniradicular mature teeth with a single canal were collected, cleaned, and stored in saline solution prior to the experiment.

The tooth crowns were removed using a disc bur with water, producing canals 14 mm in length. The working length was determined by inserting a K-file #10 until the tip was visible at the apex, and it was then set to 1 mm shorter than the total canal length.

The canals were prepared using rotary files 15/.03, 20/.05, 25/.06, 30/.06, 35/.06, and 40/.04 (Plex-V, Orodeka File, LTD, China) to the designated working length, following the manufacturer’s instructions. Between each instrument, 3% sodium hypochlorite was used as the irrigant, and 2 mL of 5% sodium thiosulfate was applied to inactivate residual NaOCl [11]. Subsequently, 2 mL of 17% ethylenediaminetetraacetic acid (EDTA) was used to remove the smear layer, followed by distilled water as the final irrigant. The teeth were then autoclaved at 121°C for 15 minutes under 26 psi.

To facilitate handling, each root was fixed with silicone inside a glass tube vial (13 × 75 mm), ensuring that the canal orifice remained free of silicone and leaving a standardized space for broth in all tubes. The entire assembly was then sterilized in an autoclave and maintained under sterile conditions until it was used.

### 2.2 Bacterial Culturing

A 0.5 McFarland concentration of *E. faecalis* was generated, and each root canal was filled with 100 µL of the suspension. To generate biofilm, the specimens were cultured for 30 days at 37°C in an aerobic environment, with the medium renewed every two days with fresh medium.

### 2.3 Study Design

The samples were randomly divided into six experimental groups (*n* = 10). The sample size of *n* = 10 per group was chosen based on a previously published statistical model in a related study by Moradi *et al*. (2022), which targeted *E. faecalis* biofilms using light-based antimicrobial methods. Their power analysis, with α = 0.05, β = 0.2, a standard deviation of 2.5, and an effect size of 0.5, indicated a required sample size of *n* = 9 per group. Therefore, using *n* = 10 in our study was considered appropriate to maintain statistical power and ensure valid comparisons across groups [12].

One group consisted of teeth irrigated only with distilled water (Group 1), while the other teeth were treated with 3% NaOCl and 17% EDTA as part of Conventional Chemomechanical Debridement (CCMD). Ten teeth were left without any additional treatment (Group 2), and the remaining teeth underwent additional laser disinfection treatments as follows:

## 3 DISINFECTION WITH PAD WITH LASER OF 450 NM **(GROUP 3)**

A diode laser (Wiser 3, Doctor Smile, Italy) with a 200 µm diameter optic fiber was used to activate a 0.1% riboflavin solution inserted into the root canals. The laser operated at a wavelength of 450 nm, with a peak power of 3 W, a frequency of 6.25 kHz, an average power of 0.8 W, and an energy density of 48 J. Following the manufacturer’s instructions, a circular movement of 1 mm/s was applied from the apex toward the coronal region. This procedure was repeated five times, with a 5-second pause between repetitions. The total duration of intracanal activation was 60 seconds, and the laser activation parameters and tip movements were consistent across all laser treatment groups.

## 4 DISINFECTION WITH PAD WITH A LASER OF 635 NM (GROUP 4)

A diode laser, set at 635 nm, with a peak power of 3 W and a frequency of 6.25 kHz, and an average power of 0.3 W, was used to activate a photosensitizing solution of toluidine blue O (TBO) at a concentration of 0.1 mg/mL.

## 5 DISINFECTION WITH (CLE) OF 808 NM (GROUP 5**)**

A diode laser activated at 808 nm, with a peak power of 3 W and a frequency of 6.25 kHz, yields a total average power of 0.8 W directly in the canal, without the use of any photosensitizing solution.

## 6 DISINFECTION WITH (CLE) OF TRIPLE WAVELENGTH (GROUP 6)

A diode laser with wavelengths of 450 nm (average power 0.1 W, peak 0.3 W), 635 nm (average power 0.2 W, peak 0.3 W), and 808 nm (average power 0.6 W, peak 2.5 W) was used at a frequency of 6.25 kHz, with a total average power of 0.9 W, also without any photosensitizing solution.

After completing the disinfection protocol, any remaining NaOCl was neutralized by rinsing the canal with 5% sodium thiosulfate, followed by rinsing with distilled water and drying with a paper point. To collect bacterial biofilm in dentin areas inaccessible to paper points, the root canals were prepared using #35 Hedstrom files [13]. Then, #40 paper points were used to transfer the solution and dentin chips into a laboratory tube containing 500 µL of nutrient broth, which was vortexed for 20 seconds to ensure thorough mixing. The sample underwent 10-fold serial dilution in distilled water across multiple tubes. Subsequently, 100 µL of the diluted solution were cultured and incubated at 37°C for 24 hours. Colony-forming units (CFUs) were counted using a magnifying lens and then converted based on the dilution factors. The entire procedure was performed under a biological hood.

### 6.1 Statistical Analysis

The data were analyzed using SPSS version 26 (2019). Prior to analysis, normality was assessed using the Shapiro-Wilk test. A *p*-value greater than 0.05 indicates a likely normal distribution, while a *p*-value ≤ 0.05 indicates deviation from normality. The Shapiro-Wilk test results were:

Before Treatment: p = 6.84 × 10^-13^

After Treatment: p = 7.68 × 10^-16^

Both *p*-values are far below 0.05, indicating that the null hypothesis of normality is rejected, and the data are not normally distributed (Table **1**).

Since the data were not normally distributed, non-parametric tests were used. The Kruskal-Wallis test assessed significant differences among groups, with *p* < 0.05 considered statistically significant.

## 7 RESULTS

The scanning electron microscope image in Fig. (**1**) shows the development of *E. faecalis* biofilm on the root canal dentin walls after 30 days of incubation.

The Kruskal-Wallis test revealed significant differences in colony-forming units (CFUs) among the groups after treatment (*p* < 0.001), as presented in Table **2**. The mean ranks for CFUs after treatment were: Group 1 (55.50), Group 2 (34.50), Group 3 (34.35), Group 4 (30.25), Group 5 (14.85), and Group 6 (13.55) (Table **3**).

The *p*-value for CFUs before treatment was 0.554, indicating no significant differences among groups. After treatment, the *p*-value was 0.000, showing significant differences (*p* < 0.05) among treatment groups. All treated groups differed significantly from the control group, particularly Groups 5 and 6.

## 8 DISCUSSION

*E. faecalis* is the most frequently encountered species in endodontic infections and plays a major role in the development of persistent periradicular lesions following root canal treatment [6]. The use of disinfecting chemicals in root canals is a primary and essential step for eliminating microorganisms from the root canal system, dentinal tubules, and periapical region [14]. Mechanical instrumentation is the most common method for reducing bacteria in infected root canals; however, complete elimination of microorganisms remains challenging. Multiple studies have shown that chemomechanical methods alone are insufficient to fully eradicate bacteria from diseased root canals [15].

Paper points or dentin chips collected from the canal walls can be used to obtain samples from within the root canal, with both methods considered viable options. While paper point sampling has been commonly used in several studies due to its simplicity, it primarily collects specimens of intracanal fluid containing planktonic microorganisms [16]. In this study, we collected dentin chips because they allow sampling of biofilm-like formations adhering to the canal walls, including microorganisms that have penetrated deeply into the dentinal tubules, using a Hedstrom file [16, 17].

A key objective of modern clinical microbiology is to develop innovative strategies that can effectively reduce the prevalence of biofilm infections in the walls of the affected root canals [18].

In Group 4, which used TBO, diode lasers were utilized as PAD. The result demonstrates a non-significant decrease, with a p-value of 0.219, in the number of colony-forming units (CFUs) compared to Group 2, which used conventional irrigation without laser. This finding aligns with the study conducted by Tennert *et al*. (2015), which investigated the effect of a 635 nm diode laser on *E. faecalis* bacteria in single-rooted teeth. When irrigation with either citric acid or EDTA was combined with PAD, the antibacterial effects were enhanced compared to using a single-irrigant protocol [19].

Nevertheless, there is still no definitive and clear identification of the actual bactericidal action at various depths of different lasers, and numerous findings present conflicting outcomes. The study conducted by Meire *et al*. (2012) evaluated the efficacy of Er: YAG and Nd:YAG lasers in combination with traditional laser irradiation for photoactivated therapy. Additionally, traditional sodium hypochlorite was used to eradicate *E. faecalis*. The investigation revealed that sodium hypochlorite demonstrated the highest efficacy in eradicating *E. faecalis*, while Er: YAG laser disinfection significantly reduced the number of living cells. Both the utilization of PAD and Nd: YAG led to a modest decrease in the quantity of *E. faecalis* [20].

The reason for using both chemical irrigation and diode laser irradiation in this study is their synergistic effect, meaning that their combined use is more effective than when applied separately. Chemical irrigation employs agents such as EDTA to dissolve the smear layer, thereby enhancing the permeability of dentinal tubules [21].

Several preliminary comparative studies have demonstrated the critical importance of irrigation with sodium hypochlorite for effective decontamination [20, 22, 23]. One study investigated the combined use of sodium hypochlorite and EDTA to develop a laser-assisted disinfection method. This approach, which integrates conventional irrigation with laser irradiation, achieves significantly improved success rates [24]. The results of the present study support these findings, with a *p*-value of 0.000. When we used an 810 nm laser in conjunction with CCMD, we obtained a mean CFU number of 40. The data, as supported by De Souza *et al*.'s (2008) study, which evaluated the standard chemomechanical approach utilizing 0.5% NaOCl and 17% EDTA, enhanced by a diode laser with an 830 nm wavelength, were also supported. Disinfection of dentin was markedly improved by diode laser application, resulting in complete eradication of bacteria in the laser Group, which may be due to the use of a high power level (3 W) compared to our 0.8 W power. This led to 98.39% eradication in the standard chemomechanical approach Group [25].

One probable reason is that bacteria absorb laser light, which can lead to damage to the bacterial cell. This effect is likely due to the presence of black pigments, such as protoporphyrin IX, in certain bacteria, which can absorb specific wavelengths in the near-infrared spectrum [10]. The damage occurs at the cellular membrane level, inducing osmotic changes that ultimately result in cell death [26].

Gram-positive bacteria, such as *E. faecalis*, exhibit greater resistance to desiccation than Gram-negative bacteria, such as *E. coli*, due to the composition of their cell wall [27]. The bactericidal process of near-infrared lasers in this case appears to necessitate the existence of oxygen in the bacterial environment [27].

The other proposed mechanism suggests that the laser light is absorbed by the dentin substrate to which bacteria are attached. Consequently, the substrate heats up, causing a localized temperature rise high enough to kill the microorganisms [10]. This may explain the study's findings, which showed the lowest CFU counts when using three laser wavelengths simultaneously: 450 nm, 635 nm, and 810 nm, all applied concurrently (*p* = 0.000).

Romeo *et al*. (2014) assessed and compared the antibacterial effects of the 532 nm (KTP laser) and the 980 nm diode laser on *E. faecalis* biofilms, in conjunction with traditional root canal operations. The use of a chemomechanical protocol involving irrigation and laser irradiation resulted in a significant reduction in bacterial presence, exceeding 96 percent for the KTP laser and 93 percent for the 980 nm diode laser. In contrast, conventional endodontic techniques achieved a reduction of approximately 67 percent. These findings confirm the efficacy of laser as an adjunctive tool for disinfection in endodontic procedures [28].

## CONCLUSION

The findings of this study highlight the persistent challenge of eliminating *E. faecalis* from the root canal system. While traditional chemomechanical methods play a crucial role in bacterial reduction, complete eradication remains difficult. Laser-assisted techniques, particularly when combined with chemical irrigation, demonstrate enhanced antibacterial effects. Our results align with previous studies, highlighting the synergistic effect of diode lasers and irrigation agents, such as sodium hypochlorite and EDTA, in enhancing bacterial reduction.

Notably, combining multiple laser wavelengths (450 nm, 635 nm, and 810 nm) yielded the greatest reduction in *E. faecalis* colonies. This suggests that photothermal and photodynamic effects contribute to the disruption of bacterial biofilms within dentinal tubules. However, variability in laser efficacy due to differences in power settings and bacterial absorption properties should be taken into account.

Overall, this study supports the integration of diode laser irradiation with conventional irrigation protocols to enhance root canal disinfection. Future research should focus on optimizing laser parameters and exploring additional antimicrobial strategies to improve treatment outcomes in endodontic infections further.

## STUDY LIMITATIONS

This study was conducted entirely under *in vitro* conditions. Therefore, further *in vivo* investigations are required to confirm the clinical relevance of the findings. Additionally, the study focused on a single bacterial species, highlighting the need for future research involving multiple microorganisms.

## Figures and Tables

**Fig. (1):**
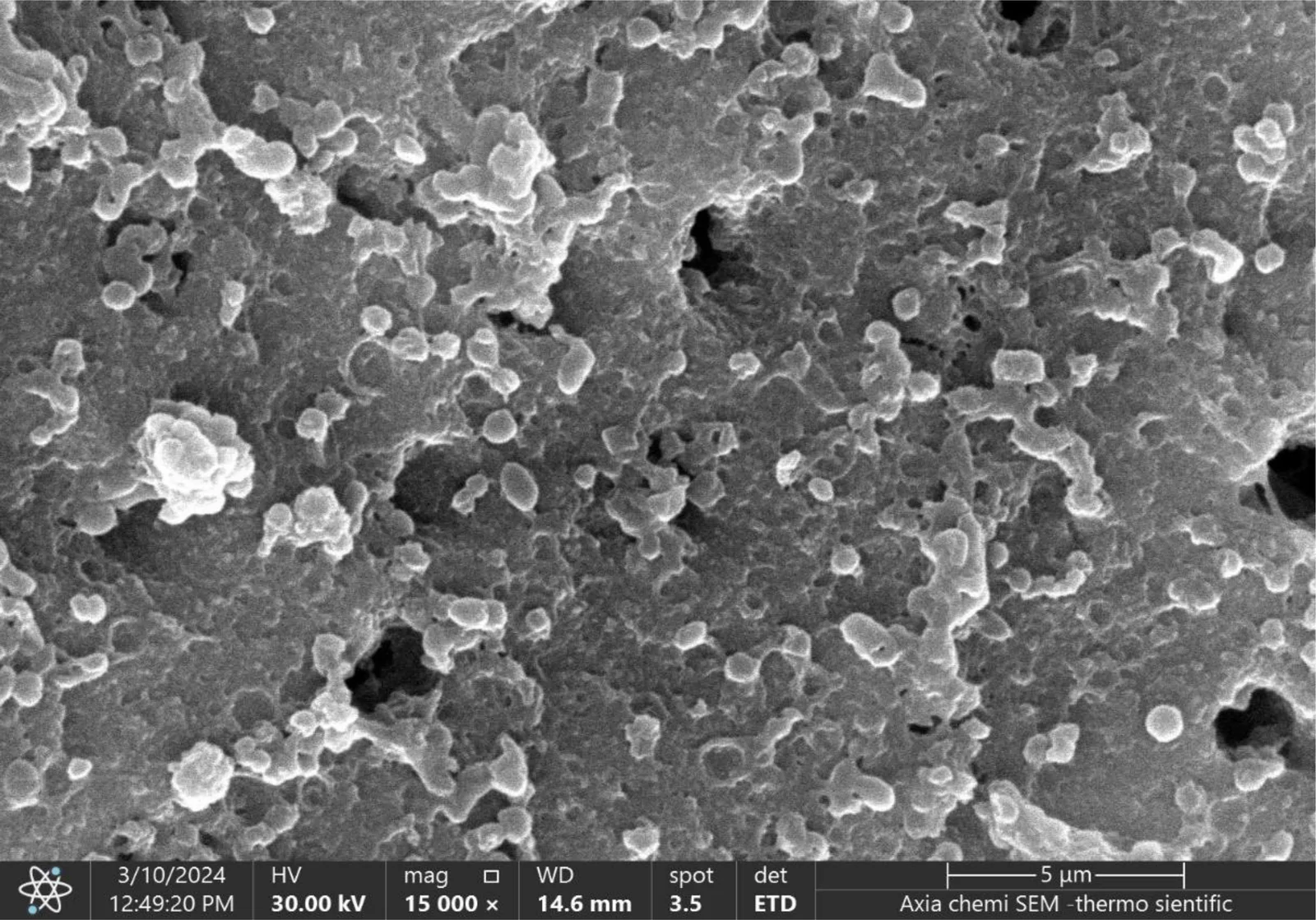
Scanning electron microscopy image shows infected root canal after 30 days of *Enterococcus faecalis* at X15000 magnification.

**Table 1:** Descriptive statistics showing the mean and standard deviation, and the minimum and the maximum value of colony-forming units before and after the treatment.

**Descriptive Statistics**					
**-**	**N**	**Mean**	**Std. Deviation**	**Minimum**	**Maximum**
Colony Forming Units Before treatment	60	6368566666666.67	13134954938360.78	20000000000	80000000000000
Colony Forming Units After treatment	60	331655.48	1382219.478	3	10000000

**Table 2:** Showing the Kruskal-Wallis test of colony forming units before and after the treatment procedure.

**Test Statistics^a,b,c^**		
**-**	**Colony Forming Units Before treatment**	**Colony Forming Units After treatment**
Kruskal-Wallis H	3.971	39.012
df	5	5
*P*-value	0.554	0.000

**Note:** ^a^ Kruskal-Wallis Test; ^b^ Grouping Variable: sample number; ^c^ Some or all exact significances cannot be computed because there is insufficient memory.

**Table 3:** Kruskal-Wallis test means ranks of colony-forming units of different groups before disinfection treatment and after disinfection treatment.

**-**	**Group Number**	**N**	**Mean Rank**
Colony Forming Units Before treatment	Group 1	10	30.50
	Group 2	10	25.90
	Group 3	10	32.75
	Group 4	10	39.05
	Group 5	10	28.50
	Group 6	10	26.30
Colony Forming Units After treatment	Group 1	10	55.50
	Group 2	10	34.50
	Group 3	10	34.35
	Group 4	10	30.25
	Group 5	10	14.85
	Group 6	10	13.55

## Data Availability

The data and supportive information are available within the article.

## LIST OF ABBREVIATIONS

CCMD: Conventional Chemomechanical Debridement
Cfus: Colony Forming Units
CLE: Conventional Laser Endodontics
PAD: Photoactivated Disinfection
